# Nonalcoholic Fatty Liver Disease and Chronic Kidney Disease: Epidemiology, Pathogenesis, and Clinical and Research Implications

**DOI:** 10.3390/ijms232113320

**Published:** 2022-11-01

**Authors:** Amedeo Lonardo, Alessandro Mantovani, Giovanni Targher, Gyorgy Baffy

**Affiliations:** 1 Division of Internal Medicine, Department of Biomedical, Metabolic and Neural Sciences, University of Modena and Reggio Emilia, 41126 Modena, Italy; 2 Section of Endocrinology, Diabetes and Metabolism, Department of Medicine, University of Verona, 37126 Verona, Italy; 3 Department of Medicine, VA Boston Healthcare System, Harvard Medical School, Boston, MA 02130, USA

**Keywords:** NAFLD, chronic kidney disease, CKD, subclinical portal hypertension

## Abstract

Nonalcoholic fatty liver disease (NAFLD) has become the most common cause of chronic liver disease worldwide, affecting up to ~30% of adult populations. NAFLD defines a spectrum of progressive liver conditions ranging from simple steatosis to nonalcoholic steatohepatitis (NASH), cirrhosis, and hepatocellular carcinoma, which often occur in close and bidirectional associations with metabolic disorders. Chronic kidney disease (CKD) is characterized by anatomic and/or functional renal damage, ultimately resulting in a reduced glomerular filtration rate. The physiological axis linking the liver and kidneys often passes unnoticed until clinically significant portal hypertension, as a major complication of cirrhosis, becomes apparent in the form of ascites, refractory ascites, or hepatorenal syndrome. However, the extensive evidence accumulated since 2008 indicates that noncirrhotic NAFLD is associated with a higher risk of incident CKD, independent of obesity, type 2 diabetes, and other common renal risk factors. In addition, subclinical portal hypertension has been demonstrated to occur in noncirrhotic NAFLD, with a potential adverse impact on renal vasoregulation. However, the mechanisms underlying this association remain unexplored to a substantial extent. With this background, in this review we discuss the current evidence showing a strong association between NAFLD and the risk of CKD, and the putative biological mechanisms underpinning this association. We also discuss in depth the potential pathogenic role of the hepatorenal reflex, which may be triggered by subclinical portal hypertension and is a poorly investigated but promising research topic. Finally, we address emerging pharmacotherapies for NAFLD that may also beneficially affect the risk of developing CKD in individuals with NAFLD.

## 1. Background

Nonalcoholic fatty liver disease (NAFLD), which is strongly and bidirectionally associated with metabolic syndrome and its individual features, encompasses a spectrum of liver diseases ranging from nonalcoholic fatty liver (NAFL) to nonalcoholic steatohepatitis (NASH) and carries the risk of developing cirrhosis and hepatocellular carcinoma in a proportion of cases [1]. These severe liver-related outcomes usually result from the progression of NAFLD diagnosed as NASH, although a subgroup of NAFLD patients diagnosed as NAFL may also develop liver fibrosis, indicating a propensity for progression [1]. To date, NAFLD has become the most common chronic liver disease worldwide, affecting up to ~30% of adults in the general population, up to ~70% of patients with type 2 diabetes (T2D), and almost all patients with severe obesity [2,3,4,5,6]. The pathogenesis of NAFLD involves a profound perturbation of metabolic homeostasis; a reprogramming of the interplay between hepatocytes, sinusoidal endothelial cells, and hepatic stellate cells; a rearrangement of the liver immune landscape; and a remodeling of the hepatic microvasculature and stromal microenvironment [7]. A key effect of persistent metabolic stress on the liver is the activation of hepatic stellate cells and the development of liver fibrosis, which typically dictates the natural course of NAFLD [8,9]. New insights into liver cell–cell interactions will define the molecular pathways that culminate in hepatic fibrogenesis and identify novel targets to interrupt disease progression to adverse clinical outcomes [10].

Chronic kidney disease (CKD) is the shared outcome of a variety of etiologies that impair the functional renal reserve, resulting in a gradual loss of function [11] and eventually causing end-stage renal disease in a subset of individuals [11]. The global estimated prevalence of CKD is approximately 10% [12,13,14,15], resulting in nearly 1.2 million deaths and 28 million years of life lost each year [12]. In high-income countries, CKD tends to be most often associated with metabolic disorders, such as T2D, obesity, and hypertension [16,17]. In low-income and middle-income countries, infectious diseases and environmental toxins are also commonly associated with CKD [18]. Given the close interconnection of CKD with metabolic disorders, the prototypical examples of pathophenotypes in NAFLD-related CKD may be diabetic nephropathy and nephropathy in the obese. The former entity features the thickening of the glomerular basement membrane, the fusion of foot processes, the loss of podocytes with the denuding of the glomerular basement membranes, and mesangial matrix expansion [19]. The latter entity exhibits glomerular hypertrophy, focal dilatation of the afferent arteriole and glomerular perihilar capillaries, and perihilar segmental sclerosis; mildly increased mesangial matrix and the thickening of glomerular basement membranes (i.e., diabetes-like features), interstitial fibrosis, foot process effacement, and hypertrophy of podocyte cell bodies may also occur [20]. These features classically characterize the most severe cases of CKD. To date, however, there are no systematic studies evaluating kidney histology among patients with NAFLD and, usually, in such cases a reference is rather made to the functional classification of CKD stages based on the estimated glomerular filtration rate (eGFR) and proteinuria, both in clinical practice and in the research arena [11].

The spectrum of similarities between NAFLD and CKD is impressive: both are common conditions (implying that their association is probable), both exhibit sexual dimorphism [21], and both are associated with an increased risk of major cardiovascular events [22]. Interestingly, the metabolic pathways, cellular phenomena, and molecular mediators involved in NAFLD and CKD are similar to each other and include insulin resistance, ectopic fat deposition, and the activation of the insulin/PI3K/Akt/mTOR and transforming growth factor-β pathways [20,23]. However, conflicting with these similarities, in both clinical and research settings NAFLD is either diagnosed based on liver histology or with imaging techniques and serum surrogate biomarkers, while CKD is most often defined functionally (by decreased eGFR and proteinuria). Despite such methodological discrepancies, the association between NAFLD and CKD has been evaluated from various perspectives since 2008, spanning from epidemiology to pathophysiology and clinical implications. 

Our narrative review aims to critically appraise this growing body of literature and highlight key areas in need of further investigation.

## 2. Epidemiology

NAFLD is a disease with heterogeneous manifestations and variable clinical outcomes [24]. The leading cause of mortality in patients with NAFLD is cardiovascular disease, followed by extrahepatic cancers and liver-related complications [25,26,27], although in other prospective studies the ranking of these causes may vary [28]. In 2008, three pioneering reports initiated the era of epidemiological research on the relationship between NAFLD and the risk of CKD. In a cross-sectional study of 2103 Italian outpatients with T2D, Targher et al. reported that NAFLD (as detected by ultrasonography) was associated with an approximately 1.9-fold increase in the risk of CKD stage ≥3 (i.e., defined as an eGFR ≤60 mL/min/1.73 m^2^ with or without coexisting overt proteinuria), independent of age, sex, smoking, adiposity measures, hypertension, diabetes duration, hemoglobin A1c, plasma lipids, and the use of antihypertensive, glucose-lowering, lipid-lowering, or antiplatelet medications [29]. In a subsequent cohort study, the same investigators monitored the occurrence of CKD stage ≥3 among 1760 outpatients with T2D and normal kidney function for 6.5 years. They found that ultrasound-detected NAFLD was associated with a moderately increased risk of incident CKD stage ≥3 (adjusted hazard ratio (HR), 1.49; 95% CI 1.1–2.2), even after adjusting for age, sex, diabetes-related factors, the use of medications, and common risk factors for CKD [30]. In another study, Chang et al. prospectively assessed the association between NAFLD and the risk of incident CKD in a cohort of 8329 South Korean men with normal kidney function and no proteinuria at baseline [31]. Over a mean follow-up of 3.2 years, these authors found that ultrasound-detected NAFLD was significantly associated with an increased incidence of CKD stage ≥3, even after adjustment for age, plasma lipid profile, and baseline eGFR (adjusted HR, 1.55; 95% CI 1.23–1.95) [31]. 

The three reports discussed above [29,30,31] have initiated a robust line of research with many epidemiological studies [15,32,33,34] and large meta-analyses [35,36]. The abundance of observational cohort studies accumulating over time has recently prompted Mantovani et al. to perform an updated meta-analysis that included 13 longitudinal cohort studies for a total of 1.2 million middle-aged individuals (28.1% of whom had NAFLD). These authors confirmed that NAFLD (as detected by blood biomarkers/scores, International Classification of Diseases codes, imaging techniques, or liver biopsy) was significantly associated with an increased risk of incident CKD stage ≥3 (pooled random-effects HR, 1.43; 95% CI 1.33–1.54) over a median follow-up of nearly 10 years, independent of age, sex, obesity, hypertension, T2D, and other common renal risk factors [37]. Importantly, in this meta-analysis, the HRs for incident CKD were substantially similar when the authors stratified the eligible studies by country [37], dispelling early concerns that the association between NAFLD and incident CKD could be limited only to Eastern countries [38]. Finally, in this meta-analysis the risk of incident CKD seemed to increase in parallel with the underlying severity of NAFLD, especially with the stage of hepatic fibrosis. This finding was in line with previous meta-analyses supporting the existence of a strong relationship between the severity of NAFLD (especially at a higher fibrosis stage) and the risk of developing liver-related complications and other extrahepatic complications, such as adverse cardiovascular outcomes and T2D [27,39,40]. Moreover, this is a key finding that agrees with a robust line of research to be further discussed in Section 3.4.

A recent umbrella review examined the associations of NAFLD with the risk of developing CKD stage ≥3 and other extrahepatic adverse outcomes [41]. Notably, the authors reported a hazard risk for incident CKD (pooled random-effects HR, 1.42; 95% CI 1.33–1.52) that was identical to that reported by the meta-analysis by Mantovani et al. [37]. This increased NAFLD-related CKD risk was consistent, even after stratification by sex (HR 1.51 for women and HR 1.49 for men) or obesity status (HR 1.21 for nonobese patients and HR 1.30 for obese patients). However, it should be noted that although the evidence available so far shows that NAFLD is associated with an increased risk of incident CKD, and such risk parallels the underlying severity of liver disease (mainly higher stages of liver fibrosis), it is not possible to establish the causality of this association because of the observational designs of the published studies. 

## 3. Pathogenesis

NAFLD is determined by a multitude of complex interactions between genetic and environmental factors. In NAFLD, pathological crosstalk occurs between the liver and kidneys and may involve other organs, such as the adipose tissue, skeletal muscles, and gut microbiota [33,42,43,44,45]. Both pathogenic determinants and epidemiological features of NAFLD are firmly embedded in the metabolic dysfunction associated with obesity, insulin resistance, and T2D. All these metabolic disorders may, at the same time, contribute to the development of CKD via several well-characterized molecular and cellular mechanisms. As alluded to above, there is now increasing evidence that the severity of NAFLD (especially when featuring more advanced stages of liver fibrosis) predicts the development and progression of CKD. Epidemiological studies also indicate that NAFLD is associated with a higher risk of incident CKD, even after adjusting for T2D, obesity, hypertension, and other major cardiorenal risk factors [37], thereby suggesting that NAFLD-associated CKD might involve some unique mechanisms. Indeed, the hepatic and systemic vasoregulatory changes seen in NAFLD may evoke the hepatorenal reflex and impair renal function. However, the relative roles of each of these risk factors is difficult to decipher in individual patients, and in clinical practice such risk factors tend to overlap in the same individuals. Illustrating this notion, a recent large cross-sectional study enrolling 13,915 Chinese individuals showed that the presence of T2D and an increased risk of liver fibrosis (assessed by noninvasive fibrosis scores) had an additive interaction on CKD incidence in NAFLD [46]. 

### 3.1. Genetic Polymorphisms

As reported in Table 1, observational studies show that the rs738409 C > G p.I148M variant in the patatin-like phospholipase domain-containing protein-3 (*PNPLA3)* gene and some other genetic polymorphisms related to a greater susceptibility to NAFLD and NASH may also play a role in the development and progression of kidney damage in both adults and adolescents [47,48,49,50,51,52,53,54,55,56,57]. For instance, in a cross-sectional study of 157 middle-aged Italian patients with T2D who underwent liver ultrasonography and vibration-controlled transient elastography for the diagnosis and staging of NAFLD, Mantovani et al. reported that the presence of rs738409 C > G p.I148M in the *PNPLA3* gene was closely associated with lower eGFR levels and a higher risk of CKD (adjusted odds ratio (OR), 6.65; 95% CI 1.65–26.8), even after adjusting for common renal risk factors and liver disease severity [53]. Notably, the authors also found that PNPLA3 mRNA expression was greatest in the liver and in the renal cortex and that renal podocytes showed high PNPLA3 mRNA and protein levels, which were comparable with the levels seen in hepatocytes and hepatic stellate cells [53]. These findings have been further replicated in some cohorts of children and adolescents (Table 1). For example, Targher et al. showed that, after adjustment for age, sex, blood pressure, adiposity measures, insulin resistance, and the histological severity of NAFLD, the presence of rs738409 C > G p.I148M in the *PNPLA3* gene was associated with both lower eGFR levels and higher 24 h proteinuria in a cohort of 142 overweight children and adolescents [48]. Herein, it is important to remember that the *PNPLA3* gene is predominantly expressed in the liver, and the G allele of rs738409 is associated with the loss of the hydrolyzing function of the protein, resulting in the accumulation of lipid droplets in hepatocytes [58]. To date, the precise mechanisms underlying the association between the *PNPLA3* genetic variant and decreased kidney function remain unclear. However, preliminary experimental data suggest that *PNPLA3* may also be expressed in both sinusoidal pericytes [59] and podocytes [53]. Hence, it is possible to hypothesize that the presence of rs738409 C > G p.I148M in the *PNPLA3* gene (resulting in reduced phospholipase activity of the enzyme) may contribute to the activation of renal pericytes, thus leading to the development of kidney steatosis and fibrosis.

To date, the only useful strategy for studying the causal nature of an association using observational data is to also recreate the randomization technique in the observational context. Mendelian randomization (MR) is an analytic research method that provides an answer to this need. MR analysis is mainly based on the assumption that a genotype is fixed before birth and genetically predicted exposure is minimally affected or unaffected by reverse causation or confounders [60]. In this regard, Park et al. conducted a complex single-variant MR study with the *PNPLA3* rs738409 variant as the genetic instrument for NAFLD to evaluate the causal effect of NAFLD on the risk of having kidney damage [61]. Interestingly, the authors showed that NAFLD was significantly associated with lower eGFR levels, even after an adjustment for coexisting metabolic disorders, thus supporting the notion that NAFLD may play a causal role in the risk of developing lower eGFR levels and incident CKD [61]. 

### 3.2. Adipose Tissue

It is universally recognized that adipose tissue is an active endocrine organ with fundamental metabolic properties in health and disease states. The principal physiological functions of white adipose tissue include body weight homeostasis, glucose and lipid metabolism, appetite regulation, reproduction, immunity, angiogenesis, fibrinolysis/coagulation, and vascular tone control [62]. In the specific setting of the possible link between NAFLD and CKD, several authors have postulated that an expanded and inflamed adipose tissue (the so-called “adipositis”) may contribute to development of microvascular disease, eventually culminating in overt CKD in a subset of individuals, possibly through a variety of pathomechanisms that include increased oxidative stress, lipotoxicity, insulin resistance, the activation of the renin–angiotensin system, and the increased release of free fatty acids and multiple proinflammatory cytokines as well as the decreased production of anti-inflammatory adipokines, such as adiponectin and leptin [33,42,44]. These putative pathomechanisms linking NAFLD to the risk of CKD have been extensively reviewed elsewhere [33,42,44].

### 3.3. Gut

The gastrointestinal tract, with its commensal microorganisms, can modulate liver–kidney crosstalk through a variety of molecular and cellular mechanisms. NAFLD is closely associated with excess caloric intake and other dietary changes that have a direct impact on the composition and function of gut microbiota. The disruption of the intestinal barrier and increased intestinal permeability due to dysbiosis may promote the entry into the portal flow of several microbial components and metabolites with various pathological effects [63,64,65]. Intestinal dysbiosis may increase the circulating levels of proinflammatory derivatives such as endotoxin (LPS) and other pathogen-associated molecular patterns, alter the metabolism and enterohepatic circulation of bile acids, impair the synthesis of short-chain fatty acids, and interfere with the farnesoid X receptor (FXR) and fibroblast growth factor (FGF)-15/19 pathways and glucagon-like peptide-1 release as well as increase the production of uremic toxins and a variety of volatile organic compounds such as indole, cresol, and phenylacetate [65,66]. Many of these changes may adversely affect the normal physiology of the liver, kidneys, and adipose tissue. The fermentation of carbohydrates and proteins by gut bacteria also yields nitric oxide (NO) and hydrogen sulfide, which act as gaseous vasotransmitters, inducing systemic low-grade inflammation and impaired vasoregulation [63,67].

### 3.4. Liver

The liver may also contribute to the occurrence of CKD with both structural and functional changes. Attesting to the fundamental role of liver histology changes and furthering the conclusions of the above-mentioned meta-analysis by Mantovani et al. [37], An et al. conducted a longitudinal study involving 455 patients with biopsy-confirmed NAFLD who were followed for a median period of 32 months [68]. These authors reported that severe lobular inflammation (unadjusted HR, 3.35, 95% CI 1.10–9.11), severe fibrosis (unadjusted HR, 3.25, 95% CI 1.12–8.84), and severe portal inflammation (unadjusted HR, 7.73, 95% CI 2.86–22.2) were significantly associated with an increased risk of developing adverse renal outcomes (defined as a ≥50% serum creatinine increase, a <30% eGFR decrease, or an eGFR <45 mL/min/1.73 m^2^) [68]. However, after adjusting for sex, age, T2D, hypertension, and eGFR at baseline, only severe portal inflammation remained significantly associated with an increased risk of adverse renal outcomes (adjusted HR 5.88, 95% CI 1.87–18.4) [68]. Additionally, convincing evidence also indicates that an inflamed and fibrotic liver may exacerbate systemic/hepatic insulin resistance and release a variety of proinflammatory factors and profibrogenic molecules that may promote vascular and renal damage [33,69].

### 3.5. Skeletal Muscle

**Figure 1:**
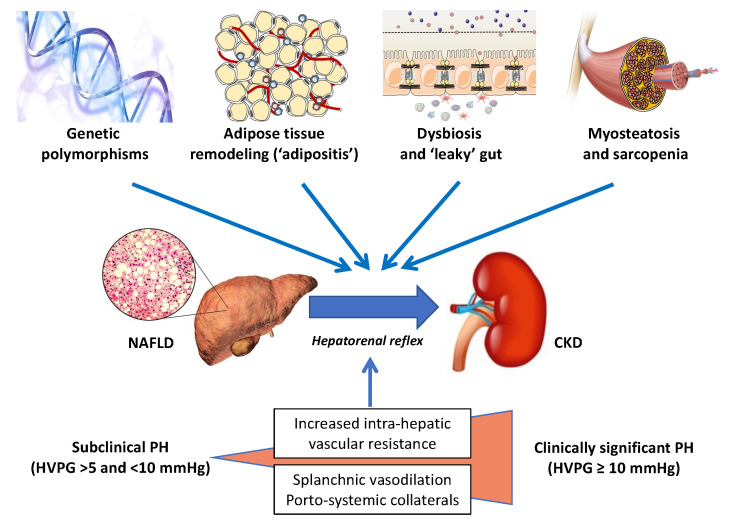
The liver–kidney axis in NAFLD. A schematic illustration of the chief “players” orchestrating the crosstalk between NAFLD and CKD. Specific genetic polymorphisms, including the patatin-like phospholipase domain-containing protein-3 (*PNPLA3)* linked to the development of NAFLD, have been also shown to predict the risk of CKD. The risk of kidney damage associated with these genetic polymorphisms is further amplified by environmental factors (e.g., obesity). By far the most prevalent of the modifiable lifestyle factors is obesity, usually originating from excessive caloric intake and reduced physical activity, which is associated with adipose tissue expansion and remodeling. These changes in the adipose tissue result in local and systemic low-grade inflammation that may promote hepatic de novo lipogenesis and steatosis by providing substrates (high levels of LCFAs) and by altering the adipokine milieu (e.g., decreased plasma adiponectin levels). Hepatic steatosis leads to hepatic insulin resistance (facilitated by diacylglycerols and ceramides), NASH, Kupffer cell activation, and the release of multiple proinflammatory cytokines and hepatokines. Myosteatosis is a metabolic dysfunction of skeletal muscles, which may contribute to peripheral insulin resistance and progress to sarcopenia, thereby promoting proteolysis and muscle breakdown. Intestinal dysbiosis is another important link between unhealthy dietary habits and physical inactivity, possibly through the increased production of a variety of microbial metabolites (e.g., SCFAs and lipopolysaccharide), hepatotoxins (e.g., ethanol), and nephrotoxins (e.g., hippuric acid, phenylacetic acid, TMA, cresol, and indole). The hepatic metabolism of some of these compounds (e.g., TMA, cresol, and indole) may further increase the nephrotoxic burden. The progression of NAFLD to advanced fibrosis and cirrhosis may further exacerbate the pathological link between NAFLD and CKD. Finally, the mechanisms behind the hepatorenal axis, by far the least appreciated mechanisms in this very complex scenario, have been detailed in the text.

In recent years, there has been an escalating interest in the skeletal muscle–NAFLD axis, specifically in the role of sarcopenia and myosteatosis as potential risk factors for the development and progression of NAFLD [70,71,72,73,74]. For instance, in a large cohort study of 333,295 participants from the UK Biobank who were followed for a median of 10 years, Peterman-Rocha et al. reported that lower muscle mass and grip strength were independently associated with a higher risk of developing severe NAFLD [75]. On these grounds, we believe that further prospective studies should address the specific roles, if any, of both sarcopenia and myosteatosis as novel risk factors for CKD in people with NAFLD. Figure 1 schematically illustrates the key “players” that are potentially involved in the development and progression of CKD in patients with NAFLD or NASH. 

Abbreviations: HVPG, hepatic venous pressure gradient; LCFAs, long-chain fatty acids; PH, portal hypertension; SCFAs, short-chain fatty acids; TMA, trimethylamine.

### 3.6. Immune Mechanisms

Although NAFLD is primarily derived from metabolic dysfunction, it is a condition with heterogenous, multilayered, pathological processes that involve a strong immunoinflammatory dimension [76]. Key features of NASH, such as lipotoxicity, oxidative stress, hepatocellular injury, and the liver inflammatory response, are particularly prone to trigger and perpetuate a vicious circle in which the immune system plays an essential role [77]. The liver, which also serves important immunological functions, is intricately involved in innate and adaptive immune responses within a uniquely shaped anatomical niche, facilitating the maximal exposure of immune cells to antigens and pathogens of both portal and systemic origins [78,79,80]. Given that ‘metaflammation’ [81] plays a key role in progressive liver injury, it is reasonable to postulate that a hepatic environment rich in immune cells can modulate NAFLD development and severity [82]. However, our understanding of the immune pathogenesis of NAFLD is incomplete, and we are not fully aware of how specific immune cell subsets mutually interact and how they interplay with stromal liver cells during disease development and progression [77]. Even less is known about how immune-mediated molecular mechanisms may be involved in the pathologic interplay between the liver and kidney in NAFLD. 

### 3.7. Hepatorenal Reflex

This term denotes a functional connection between the liver and kidneys that becomes clinically manifest during the development of decompensated cirrhosis and its progression into refractory ascites and the so-called hepatorenal syndrome (HRS) [83]. HRS manifests as severe functional kidney failure (i.e., reversible after liver transplantation) with the activation of the renin–angiotensin system and systemic vasoconstriction but without discernible structural abnormalities in the kidneys [84]. The mechanisms underlying HRS are incompletely understood but include splanchnic vasodilation, an increased inflammatory response, and cirrhotic cardiomyopathy [85]. However, experimental evidence now suggests that early changes in portal and splanchnic vasoregulation associated with NAFLD may initiate a pathological hepatorenal reflex before culminating in HRS, which therefore represents only the “tip of the iceberg” submersed into the noncirrhotic stages of NAFLD. 

While it is likely that multiple pathophysiologic mechanisms are involved in the development of the hepatorenal reflex in NAFLD, recent research data have highlighted two main concepts that are particularly pertinent in this regard. The impairment of sinusoidal blood flow and increased intrahepatic vascular resistance (IHVR) are at the core of both concepts. Blood flow in the liver sinusoids becomes increasingly sluggish during the course of NAFLD due to external compression (i.e., hepatocellular lipid accumulation and ballooning, and the inflammatory and fibrogenic expansion of the extracellular matrix), impaired vasoreactivity (sinusoidal endothelial dysfunction and the procontractile hepatic stellate cell phenotype), and intravascular impediments (the formation of lipid emboli, the adhesion and entrapment of recruited leukocytes, microthrombosis, and neo-angiogenesis) [86]. Several vasoactive substances have been implicated in the vasoregulatory changes linking increased portal pressure and splanchnic vasodilation, such as nitric oxide, glucagon, bile salts, atrial natriuretic peptide, adrenomedullin, and endocannabinoids [87,88].

In the first concept, intrahepatic oxygen zonation is intensified when increased postprandial splanchnic oxygen consumption is compounded by impaired hepatic microcirculation due to hepatocellular swelling from lipid accumulation, resulting in hypoxia, reduced ATP production, and the increased release of adenosine, which is believed to stimulate the hepatorenal reflex, leading to reduced renal blood flow [89]. This process is aggravated by the ingestion of fructose, which is metabolized by consuming ATP, resulting in the production of more adenosine [89]. Apparently, these early hemodynamic and metabolic changes may be reverted by dietary changes or bariatric surgery procedures. 

The second concept focuses on the mechanistic role of increased portal pressure in NAFLD pathogenesis [90,91]. It has been known that the hepatorenal reflex can be provoked experimentally by inducing pneumoperitoneum in rats [92]. Following an abruptly increased portal pressure, these rodents will exhibit decreased glomerular filtration and oliguria that can be abolished by denervation [92]. However, the impact of subtle increases in portal pressure on NAFLD pathophysiology mostly remains unexplored [90], while accumulating evidence from experimental and clinical observations clearly indicates that subclinical portal hypertension, defined by a hepatic venous pressure gradient (HVPG) of 5 to 10 mmHg, may develop early in the course of NAFLD [93,94,95,96,97,98]. The molecular pathways and pathological consequences of mechanosignaling in NAFLD were summarized in a recent review [99]. With the progression of NAFLD, sustained IHVR promotes vasodilation in splanchnic and systemic arteries, resulting in decreased systemic vascular resistance and eventually in the development of spontaneous portosystemic shunts [100]. Hemodynamic measurements in experimental fatty livers indicate that unfavorable changes in hepatic circulation are accompanied by splanchnic vasodilation, arterial hyporesponsiveness to vasoconstrictors, and a decrease in mean arterial blood pressure [101], potentially jeopardizing renal blood flow.

While the phenomenon of subclinical portal hypertension in noncirrhotic NAFLD is recognized as a potential driver of cellular and molecular mechanisms, eventually leading to fibrosis, cirrhosis, and hepatocellular carcinoma [90], it is reasonable to postulate that subclinical portal hypertension may also play a role in triggering a subclinical hepatorenal reflex that, projected over time, may contribute to the development and progression of CKD. However, this intriguing hypothesis will need to be tested by specific clinical and experimental studies. To this end, the utilization of novel endoscopic ultrasound-guided techniques allowing the assessment of portal pressure through the direct access of the portal vein and hepatic vein in patients with NAFLD at various stages of disease severity [90,102] will hopefully provide a response.

## 4. Clinical Implications

The epidemiological and pathophysiological findings discussed above may have important clinical implications. 

### 4.1. Diagnosis

Based on the abundant evidence discussed in paragraphs 3 and 4, individuals with NAFLD should be always screened for CKD and should be regularly monitored for kidney function. In addition, it is important to highlight that, since NAFLD is closely inter-related with metabolic disorders (such as, for example, insulin resistance, overweight/obesity, and T2D), an international panel of experts recently proposed a change of terminology from NAFLD to metabolic dysfunction-associated fatty liver disease (MAFLD) [103]. Although the evidence regarding the concordance or even superiority of the MAFLD definition, compared to the NAFLD definition, in detecting subjects at high risk of CKD is preliminary, current data suggest that the newly proposed MAFLD definition may identify patients with CKD as accurately as the NAFLD definition [32,34]. To the extent that serum aminotransferase levels are often used for the calculation of noninvasive biomarkers of fibrosis, serum alanine aminotransferase (ALT) levels are indirectly associated with the risk of CKD. However, serum aminotransferase levels per se have not been found to be useful in CKD risk prediction. For example, in a sample of nearly 13,000 U.S. Hispanic Latino adults, Missikpode et al. [104] reported that elevated serum aminotransferase levels were not independently associated with reduced eGFR. In a cohort of 217 Chinese patients with biopsy-proven NAFLD, Sun et al. [49] found that patients with NAFLD and persistently normal ALT levels were more likely to have higher levels of glomerular and tubular damage biomarkers compared to their counterparts with abnormal ALT levels. 

### 4.2. Management

#### 4.2.1. Lifestyle Modification

Both cross-sectional and longitudinal studies support the notion that lifestyle modification has an important role in reducing the risk of CKD among individuals with NAFLD or NASH (irrespective of their body mass indexes). For example, in a population-based study of South Korean individuals, Jung et al. reported that physical exercise was associated with a lower risk of both prevalent and incident CKD (defined as eGFR <60 mL/min/1.73 m^2^ and/or overt proteinuria) in individuals with NAFLD [105]. In another cohort study of 10,311 Chinese individuals with newly diagnosed NAFLD and without CKD at baseline, Hu et al. reported that short-term weight loss of at least 7% could decrease the risk of developing incident CKD, especially among those with obesity and elevated blood pressure [106]. Although these studies were conducted in Asian countries and need to be further validated in other ethnic groups, the results are both biologically plausible and encouraging. However, it should be noted that the feasibility of substantial long-term lifestyle changes is fraught with difficulties in many patients with NAFLD [107]. 

#### 4.2.2. Pharmacotherapy

Many excellent review articles have recently been published on emerging drug approaches for the treatment of NAFLD (see, for example, among others [108,109,110,111,112]). Although a detailed discussion of the recent developments in pharmacotherapy for NAFLD/NASH is beyond the scope of our review, they are briefly summarized in Table 2. The most promising randomized controlled trials in the NAFLD arena were published since 2010 [113,114,115,116,117,118,119,120,121,122,123]. 

The fundamental concept inspiring the logic of pharmacotherapy in patients with NAFLD or NASH, who are at high risk of developing incident CKD, is to try to “kill two birds with one stone” [124]. In a comprehensive review article, Sumida et al. recently identified drug classes that could be utilized (or could be considered in the future) to this dual end. These agents include metabolic modifiers, such as peroxisome proliferator-activated receptor (PPAR)-γ agonists (i.e., pioglitazone), PPAR-α/γ agonists (saroglitazar and aleglitazar), PPAR-α/δ agonists (elafibranor), FXR agonists (obeticholic acid), antioxidants (oltipraz and bardoxolone methyl), and anti-inflammatory and antiapoptotic agents (cenicriviroc, BMS-81160, PF-04634817, and selonsertib), as well as antifibrotic agents (belapectin and GCS-100), anti-hypertensive drugs (aparerenone), prebiotics, probiotics, and symbiotics [124]. In addition, there is now increasing scientific interest in the effects of newer anti-hyperglycemic agents, such as glucagon-like peptide-1 receptor agonists (GLP-1RAs) and sodium-glucose cotransporter-2 (SGLT2) inhibitors, in patients with NAFLD or NASH, as these agents may not only exert hepatoprotective actions but also have a marked benefit on cardiovascular and renal outcomes, regardless of the presence or absence of T2D [15,34,109,125]. 

##### GLP-1 Receptor Agonists

The so-called “incretin effect” is defined as the increase in pancreatic insulin secretion after oral glucose ingestion compared with the insulin secretion after an equivalent amount of glucose administered as an intravenous glucose infusion [126]. The incretin effect mainly results from the combined action of two main incretin hormones that are produced in the gastrointestinal tract, namely gastric inhibitory polypeptide (GIP) and glucagon-like peptide-1 (GLP-1). In short, GLP-1 improves glucose disposal, mainly through the activation of its specific receptors located on pancreatic (β and δ) cells and via numerous indirect systemic effects, including reduced appetite and the ensuing weight loss (mainly via the activation of GLP-1 receptors in hypothalamic regulatory centers of hunger and satiety), the inhibition of gluconeogenesis, delayed gastric emptying, and slowed peristalsis of small intestine as well as increases in the metabolic rate, the energy consumption of brown adipose tissue cells, and peripheral adipose tissue lipolysis (mainly via the sympathetic nervous system) [127,128,129]. 

To date, licensed GLP-1RAs for the treatment of T2D include exenatide, liraglutide, lixesenatide, dulaglutide, and semaglutide, which differ mostly in the frequency of their administration, dosage, and half-life [126]. Some of these GLP-1RAs have also been evaluated in phase 2 randomized controlled trials (RCTs) to specifically treat patients with NAFLD or NASH. To assess the efficacy of GLP-1RAs for the specific treatment of NAFLD or NASH, Mantovani et al. performed a systematic review and meta-analysis of 11 placebo-controlled or active-controlled phase 2 RCTs (involving a total of 936 middle-aged overweight or obese individuals with NAFLD or NASH, the majority of whom had pre-existing T2D) that used subcutaneous liraglutide (*n* = 6 RCTs), exenatide (*n* = 3 RCTs), dulaglutide (*n* = 1 RCT), or semaglutide (*n* = 1 RCT), as detected by imaging techniques (*n* = 9 RCTs) or liver biopsy (*n* = 2 RCTs) [130]. Compared to either placebo or reference therapies, GLP-1RA treatment for a median period of 26 weeks was associated with a significant reduction in the absolute percentage of liver fat content, as assessed with magnetic-resonance-based imaging techniques (pooled weighted mean difference, −3.92%, 95% CI −6.27% to −1.56%) and improvements in serum aminotransferase levels, as well as with a greater histological resolution of NASH without a worsening of fibrosis (pooled random-effects OR, 4.06, 95% CI 2.52–6.55; for liraglutide and semaglutide only); conversely, there was no significant difference in the percentage of those with improvement in fibrosis stage without the worsening of NASH [130]. From these data, it is reasonable to assume that, if confirmed in larger phase 3 RCTs, subcutaneous liraglutide and semaglutide will become promising treatment options for NAFLD or NASH. Large RCTs and some meta-analyses also reported that treatment with GLP-1RAs markedly reduced the long-term risk of developing CKD and overt proteinuria, especially in patients with T2D [131,132,133]. 

##### SGLT-2 Inhibitors

This class of glucose-lowering agents derives from our increased understanding of the role played by the renal SGLT transporter system in preserving glucose homeostasis [134]. SGLT-2 inhibitors specifically inhibit the high-capacity, low-affinity SGLT-2 transporters in the proximal tubule of the kidney [135]. This achievement has resulted from the synthesis and evaluation of selective SGLT-2 inhibitors, given that the predecessor molecule phlorizin had severe gastrointestinal side effects resulting from the blockade of SGLT-1 in the gastrointestinal tract [134]. SGLT-2 inhibitors reduce the worsening of kidney function through various mechanisms, including improved renal oxygenation, decreased intrarenal inflammation, and curtailed glomerular hyperfiltration occurring via increased natriuresis and tubuloglomerular feedback, independent of glycemic control [134]. Given that nondiabetic CKD also exhibits single-nephron hyperfiltration and elevated albuminuria, SGLT-2 inhibitors may currently be repositioned from diabetic to nondiabetic kidney disease, and large phase 3 RCTs are currently ongoing to examine the efficacy and safety of these agents in patients with CKD owing to both diabetic and nondiabetic etiologies [133,134].

Compared to GLP1-RAs, the efficacy of SGLT-2 inhibitors in NAFLD is less well documented. In a recent meta-analysis that included 12 phase 2 RCTs with aggregate data on 850 middle-aged overweight or obese individuals with NAFLD (most of whom had coexisting T2D), Mantovani et al. reported that, compared to a placebo or reference therapy, treatment with SGLT-2 inhibitors for 24 weeks (mostly empagliflozin and dapagliflozin) was associated with significant reductions in serum liver enzyme levels as well as in the absolute percentage of liver fat content, as assessed with magnetic-resonance-based techniques (pooled weighted mean difference: −2.05%, 95% CI −2.61 to −1.48%) [136]. However, RCTs based on liver histology outcomes are eagerly awaited. Conversely, although there are no “head-to-head” RCTs, SGLT-2 inhibitors might offer greater nephroprotection than GLP1-RAs, especially in patients with T2D. In this regard, the British National Institute for Health and Care Excellence (NICE) estimated that among 1000 individuals offered usual diabetes care, 92 will develop end-stage kidney disease in a 5-year time frame, while the use of GLP1-RAs or SGLT-2 inhibitors will, respectively, provide gains of 19 and 26 fewer individuals (https://magicevidence.org/match-it/200820dist/#!/sof/data-set/adults-ckd).

##### Renin–Angiotensin System Inhibitors 

According to guidelines, patients who have CKD and/or T2D with albuminuria have a blood pressure goal set at <130/80 mmHg, and blood pressure levels exceeding this threshold will necessitate lifestyle modifications and anti-hypertensive pharmacotherapy [137]. Angiotensin-converting enzyme (ACE) inhibitors should be the drugs of first choice and angiotensin receptor blockers (ARBs) should be used if ACE-inhibitors are not well tolerated [137]. Whether renin–angiotensin system inhibitors also favorably affect NAFLD or NASH is poorly defined. However, a territory-wide cohort study of hypertensive individuals with NAFLD followed for at least 5 years found that treatment with ACE-inhibitors was significantly associated with a lower risk of liver-related events (subdistribution hazard ratio (SHR) 0.48; 95% CI 0.35–0.66), liver cancers (SHR 0.46; 95% CI 0.28–0.75), and cirrhotic complications (SHR 0.42; 95% CI 0.27–0.66), while ARB treatment was not. Notably, treatment with ACE-inhibitors was also associated with a greater reduction in liver-related events in patients with CKD than in those without kidney damage [138].

##### Modulation of Intrahepatic Vascular Resistance

The complex physiology of liver sinusoids may offer a variety of cellular and molecular targets for mitigating the adverse impact of NAFLD on intrahepatic vascular resistance (IHVR). The prevention of hepatic steatosis by lifestyle modifications aimed at reducing excess caloric intake and controlling the adversities of obesity and T2D remains the best strategy. Several drug candidates are also considered for the management of early portal hypertension, although most of these compounds have not yet entered the clinical phase, and current evidence for their potential use is based on experimental data. 

The pharmacological management of early portal hypertension associated with noncirrhotic NAFLD may target reversible changes in sinusoidal vasoregulation, leading to increased IHVR, such as the dysfunction of short-lived effector cells (SLECs) and the procontractile and profibrotic transformation of hepatic stellate cells (HSCs) [86,139]. Of the currently considered strategies, statins stand out due to their long safety record and pleiotropic effects on hepatic circulation. Statins stimulate LSEC-mediated nitric oxide (NO) release by upregulating KLF2, a transcription factor responsive to shear stress and targeting the gene of eNOS, and by inhibiting the RhoA/ROCK pathway, which promotes LSEC capillarization and the contractility of HSCs [140,141,142]. There is now substantial evidence for the beneficial impact of statins on IHVR and the survival of patients with portal hypertension [143,144,145]. In fact, the United States Preventive Services Task Force now recommends that clinicians prescribe a statin for the primary prevention of cardiovascular disease for adults aged 40 to 75 years who have one or more risk factors (i.e., dyslipidemia, T2D, hypertension, or smoking) and an estimated 10-year risk of a cardiovascular event of 10% or greater, which encompasses most patients with NAFLD. A more widespread use of statins in this patient population might also have a beneficial effect on CKD development. 

Nuclear farnesoid X receptor (FXR) agonists represent another drug class with a potential to modulate IHVR [142,146]. Obeticholic acid, a synthetic bile acid ligand of FXR, which is currently in the pipeline to be approved in NASH pharmacotherapy, reduces experimental portal hypertension by upregulating eNOS and repressing Rho-kinase activity [147]. Other steroidal and nonsteroidal FXR agonists have been shown to decrease the rate of hepatic de novo lipogenesis, reduce arachidonic-acid-induced inflammation, and inhibit HSC contractility [148,149,150,151]. Additional agents in preclinical phases, but with promising effects on HSC activation and contractility, include renin–angiotensin system inhibitors [152], mitochondrial antioxidants [153,154], and selective beta-3 receptor agonists [155,156]. However, the applicability of these and many other drug candidates to the safe and efficient management of early portal hypertension with a potential beneficial impact on the NAFLD-related development and progression of kidney injury remains to be demonstrated.

## 5. Conclusions and Research Agenda

Recent advances in epidemiological research have been aimed at altering the traditional hierarchy of evidence. Historically, systematic reviews and meta-analyses topped the pyramid, representing the strength of evidence-based medicine, while today RCTs and meta-analyses of RCTs tend to receive priority [157]. This implies that large and high-quality RCTs should be conducted regarding all aspects of the association between NAFLD and the risk of developing CKD.

**Figure 2:**
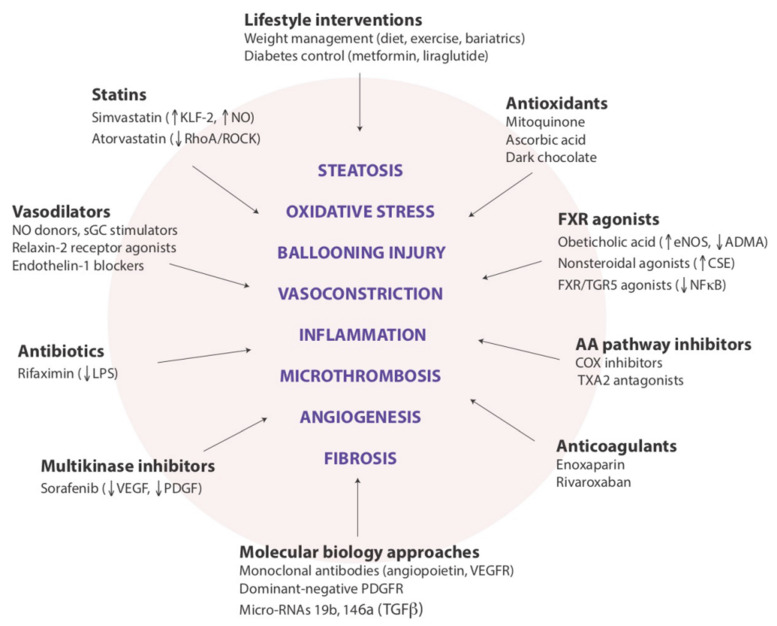
Potential targets for modulating intrahepatic vascular resistance in NAFLD.

With regard to the pathogenic links between NAFLD and CKD, in our opinion there are at least two major research points that need to be expanded by future studies. The first is to ascertain whether the skeletal muscle, which has an increasingly recognized role in NAFLD pathophysiology [74,158], also modulates the link between the liver and kidneys in both health and disease states. The second is whether the hepatorenal axis, as discussed above, represents a novel pathogenic mechanism that could become the new target of emerging pharmacotherapies aimed at reducing the risk of developing CKD among individuals with NAFLD or specifically with NASH (Figure 2).

Meanwhile, we should remain vigilant of the possibility of CKD developing in patients with NAFLD and consequently consider a mandatory periodic assessment of renal function in this patient population [15]. Finally, in the near future, the implementation of innovative methods for the measurement of portal pressure (that are less invasive than the current gold standard, HVPG) may allow for a more widespread “functional” stratification of patients with NAFLD, thereby enabling physicians and researchers to personalize follow-up and therapeutic approaches. 

Legend to Figure 2: This cartoon illustrates the variety of drug approaches to be evaluated for addressing the functional components in the pathophysiology of portal hypertension associated with NAFLD. We believe that these drug classes and examples, beyond their promising indications for preventing the progression of liver disease, may also prove to be beneficial for preventing the development of CKD among individuals with NAFLD (reprinted from Ryou et al. [102]). 

## Figures and Tables

**Table 1:** Observational studies examining the associations between specific polymorphisms related to the development and progression of NAFLD and the risk of CKD.

Author, Year, [Ref.]	Study Characteristics	NAFLD Diagnosis	Genetic Polymorphism	Creatinine-Based Glomerular Filtration Rate (GFR) Estimation Equations	Covariate Adjustment	Main Findings
Oniki et al., 2015 [54]	Cross-sectional and retrospective longitudinal studies: 740 and 393 Japanese individuals followed for 5.5 years during a health screening program for cross-sectional and longitudinal analyses, respectively	Ultrasonography	*PNPLA3* rs738409 G allele	MDRD-eGFR equation	Age, sex, BMI, T2D, hypertension, dyslipidemia, and hepatic steatosis (on ultrasound)	Carriers of G/G genotype and normal weight had lower eGFR levels than those with C/C or C/G genotypes.
Musso et al., 2015 [55]	Cross-sectional study: 202 Italian non-obese and nondiabetic individuals	Biopsy	*PNPLA3* rs738409 G allele	CKD-Epidemiology Collaboration equation	Age, sex, BMI, and metabolic syndrome	Carriers of G/G or C/G genotypes had higher risks of albuminuria and CKD than those carrying C/C genotype.
Mantovani et al., 2019 [47]	Cross-sectional study: 101 Italian postmenopausal women with T2D	FLI ≥60 (ultrasonography in a subset of patients)	*PNPLA3* rs738409 G allele	CKD-Epidemiology Collaboration equation	Age, diabetes duration, HbA1c insulin resistance, systolic blood pressure, hypertension treatment, and FLI ≥ 60	Carriers of G/G genotype had lower eGFR levels and higher prevalence of CKD than those with C/C or C/G genotypes.
Targher et al., 2019 [48]	Cross-sectional study: 142 Italian children and adolescents with NAFLD	Biopsy	*PNPLA3* rs738409 G allele	Bedside Schwartz equation	Age, sex, systolic blood pressure, measures of adiposity, insulin resistance, NASH, liver fibrosis	Carriers of G/G genotype had lower eGFR levels and higher proteinuria than those with C/C or C/G genotypes.
Marzuillo et al., 2019 [56]	Cross-sectional study: 591 Italian obese children	Ultrasonography	*PNPLA3* rs738409 G allele	Bedside Schwartz equation	Sex, the duration of obesity, ALT, insulin resistance, and plasma lipids	Carriers of G/G genotype had lower eGFR levels than those with C/C or C/G genotypes.
Di Costanzo et al., 2019 [57]	Cross-sectional study: 230 Italian overweight/obese children	Magnetic resonance imaging	*PNPLA3* rs738409 G allele	Bedside Schwartz equation	Age, sex, pubertal status, waist circumference, diastolic blood pressure, and hepatic steatosis	Carriers of G/G genotype had similar eGFR levels compared to those with C/C or C/G genotypes.
Sun et al., 2020 [49]	Cross-sectional study: 227 Chinese patients with NAFLD	Biopsy	*PNPLA3* rs738409 G allele	CKD-Epidemiology Collaboration equation	Age, sex, BMI, waist circumference, hyperuricemia, insulin resistance, hypertension, T2D, NASH, and the histologic stage of liver fibrosis	Patients with NAFLD who carried the *PNPLA3* rs738409 G allele were at higher risk of glomerular and tubular injuries.
Mantovani et al., 2020 [53]	Cross-sectional study: 157 Italian postmenopausal women with T2D	Ultrasonography and vibration-controlled transient elastography	*PNPLA3* rs738409 G allele	CKD-Epidemiology Collaboration equation	Diabetes duration, HbA1c, hypertension, the presence of significant fibrosis (on elastography), and abnormal albuminuria	Carriers of G/G genotype had lower eGFR levels and higher prevalence of CKD than those with C/C or C/G genotypes.
Koo et al., 2020 [50]	Cross-sectional study: 396 South Korean individuals with biopsy-proven NAFLD from the Boramae NAFLD study	Biopsy	*MBOAT7* rs641738 T allele; *PNPLA3* rs738409 G allele; *TM6SF2* rs58542926 T allele	CKD-Epidemiology Collaboration equation	Age, sex, BMI, and metabolic syndrome	Carriers of T/T genotype in *MBOAT7* rs641738 had a higher prevalence of CKD than those with A/T or A/A genotypes. No association was found between CKD and either the *PNPLA3* rs738409 G allele or the *TM6SF2* rs58542926 T allele.
Di Sessa et al., 2020 [51]	Cross-sectional study: 684 Italian obese children	Ultrasonography and/or ALT >40 UI/L	*HSD17B13* rs72613567 T allele	Bedside Schwartz equation	Sex, duration of obesity, *PNPLA3* rs738409 G allele; *TM6SF2* rs58542926 T allele, BMI, insulin-resistance, plasma lipids, and hepatic steatosis	Carriers of T/T genotype had lower eGFR values than those with A/T or A/A genotypes.
Baratta et al., 2022 [52]	Cross-sectional study: 538 Italian NAFLD outpatients with available renal function data	Ultrasonography	*MBOAT7* rs641738 T allele; *PNPLA3* rs738409 G allele; *TM6SF2* rs58542926 T allele; *GCKR* rs780094 T allele	CKD-Epidemiology Collaboration equation	BMI, metabolic syndrome, and liver fibrosis (as assessed by the FIB-4 index)	None of the NAFLD-associated genetic risk variants were associated with eGFR decline.

Abbreviations: ALT, alanine transferase; BMI, body mass index; CKD, chronic kidney disease; eGFR estimated glomerular filtration rate; FIB-4, Fibrosis-4; FLI, fatty liver index; GCKR, glucokinase regulatory protein; HSD17B13, 17-β hydroxysteroid dehydrogenase 13; MBOAT7, membrane-bound O-acyltransferase domain containing 7; NAFLD, nonalcoholic fatty liver disease; NASH, nonalcoholic steatohepatitis; PNPLA3, patatin-like phospholipase domain-containing 3; T2D, type 2 diabetes; TM6SF2, trans-membrane 6 superfamily member 2.

**Table 2:** Principal randomized controlled trials for NAFLD pharmacotherapy.

*Outcome/Drug*	Vitamin E [113]	Pioglitazone [113]	Obeticholic Acid [114]	Resmetirom [115]	Semaglutide [116]	Lanifibranor [117]	Aldafermin [118]	Elafibranor [119]	Cenicriviroc [120]	Selonsertib [121,122,123]
**Improvement of steatosis**	yes	yes	yes	yes	yes	NA	NA	no	NA	No
**Improvement of steatohepatitis**	yes	yes	yes	NA	yes	yes *	NA	yes	NA	yes §
**Improvement of fibrosis**	no	no	yes	NA	no	yes	no	NA	yes	no

* Significant decrease in the composite histological SAF score at the dose of 1200 mg vs. placebo. § Improved NAS/no change vs. worse. NA = not addressed.
